# Supplementary material for: Predicting treatment outcomes following an exacerbation of airways disease
Source: PLoS One. 2021 Aug 20;16(8):e0254425. doi: 10.1371/journal.pone.0254425 (PMC8378754; doi:10.1371/journal.pone.0254425)
Supplement: S1 Appendix — (DOCX) [file pone.0254425.s001.docx]

**S1 Appendix**

**Predicting Treatment Outcomes Following an Exacerbation of Airways Disease**

**Andreas Halner¹, Sally Beer², Richard Pullinger^2^, Mona Bafadhel¹, Richard E.K. Russell ^1,3^**

***1. Respiratory Medicine Unit, Nuffield Department of Medicine, University of Oxford, UK***

***2. Department of Emergency Medicine, Oxford University Hospitals NHS Foundation Trust, UK***

***3. NIHR Biomedical Research Centre, University of Oxford, UK***

***Corresponding author: Richard E. K. Russell***

***Email:*** [***Richard.Russell@ndm.ox.ac.uk***](mailto:Richard.Russell@ndm.ox.ac.uk) **(RR)**

**S1 Appendix Statistical Analysis**

In this study, random forest models were developed to predict systemic corticosteroid (SCS) physician prescription and to predict treatment failure. 1000 trees were used in a random forest model and the number of variables to be randomly selected at each split in each tree node was equal to the square root of the number of predictors being considered, as per recommendations[1]. The ‘R’ package ‘caret’ was used for random forest model development and feature elimination[2]. Variable importance scores in the SCS prescription prediction and treatment failure prediction models were calculated from the mean increase in out of bag model error when permuting the relevant variable[1]. Based on the variable model importance scores considering variables which passed the p < 0.1 univariate analysis filter, the least important predictors were iteratively eliminated and model performance calculated for each smaller predictor subset model[3]. Leave-one-out cross-validation (LOOCV) was then used to select a suitable subset of predictors and validate model performance. All random forest models using between 1 variable and the number of variables which passed the p < 0.1 univariate analysis filter needed for maximal area under receiver operating characteristic (ROC) curve performance (to nearest 0.01) were considered provided that the variable importance scores were > 0. (A negative variable importance score would indicate that a variable is uninformative and hence should not be included in the model since any apparent improvements in AUC achieved through including such a variable is likely due to overfitting). We deliberately used an initial p < 0.1 univariate filter rather than a more limiting threshold (e.g. p <0.05 or <0.03). This is because the initial filter step serves to include as many variables as possible for consideration in the multivariate model but to remove variables likely to represent noise rather than being truly informative. Using a stricter initial univariate filter threshold would bias the multivariate classifier model comparisons because the small subset of variables apparently important by virtue of meeting a p <0.05 or <0.03 threshold, for example, may actually not be the most informative predictors in the multivariate context. This would compromise our study objective of conducting an unbiased classification of patients as being prescribed SCS or not and as failing versus succeeding treatment.

Confidence intervals for the AUC are not reported due to the following: since LOOCV was used, each of the n models for SCS prescription prediction and treatment failure prediction (where n is the total sample size in the study) is based on training data with (n-1) out of the n samples in common and just one sample different. This means that the AUCs of the n models in leave-one-out cross-validation are almost identical and hence AUC confidence intervals would be misleading since the apparent confidence intervals would be much narrower compared to what would be obtained if the model were applied in completely separate cohorts.

Convenient metrics for model selection such as the Akaike Information Criterion (AIC)[4] or Bayesian Information Criterion (BIC)[5] could not be applied in the context of our random forest SCS prescription prediction and treatment failure prediction algorithms. This is because 1) by definition not all decision trees in the ensemble comprising the random forest include use of the same variables[1] and so the effective ‘number of parameters estimated by the classifier’ in the case of a random forest is difficult to define; 2) random forest model development does not use maximum likelihood. Both the number of parameters estimated by a classifier and the maximum value of the likelihood function for a model must be known in order to compute AIC[4] or BIC[5]. The primary reason why we used the random forest classifier in our study rather than using a linear parametric model such as the logistic regression is that random forests can be effective in preventing overfitting[1], even in a low sample size high dimensional context. This is especially relevant in our study since out of the 81 study participants 43 failed treatment and 38 succeeded treatment, with 64 patients given SCS and 17 patients not given SCS. This means that the maximum number of variables we could consider for inclusion using logistic regression at any point in the analysis pipeline would be 3.8 variables (10 events of minority class per variable statistical rule of thumb) in the case of treatment failure prediction and 1.7 variables in the case of SCS prescription prediction. A priori assumptions regarding which 4 variables to consider for treatment failure prediction and which 2 variables to consider for SCS prescription prediction instead of mathematically-driven selection of variables would jeopardise the discovery of the objectively best predictors of treatment failure and SCS prescription in a multivariate context. In contrast, the use of a random forest approach enables larger number of variables to be considered and optimal combinations of predictors identified (because the random selection of variables which can be considered for use in the nodes of different decision trees in the random forest ensemble counteracts potential overfitting)[1]. Therefore, the use of a random forest model was felt to be highly appealing in the context of our study.

**S1 Table | Additional comparison of D0 exacerbation characteristics of patients given SCS versus patients not given SCS.**

| **Characteristic** | | **Exacerbation** | | |
| --- | --- | --- | --- | --- |
|  | | **n=81 Events** | | |
|  |  | **SCS Given, n=64** | **SCS Not Given, n=17** | ***P*-value** |
| Male, n (%) | | 26 (41) | 8 (47) | 0.63 |
| Age (years)* | | 53 (29-68) | 51 (38-58) | 0.96 |
| Asthma, n (%) | | 46 (72) | 13 (76) | 0.84 |
| COPD, n (%) | | 18 (28) | 4 (24) | 0.75 |
| Current smokers, n (%) | | 16 (25) | 5 (29) | 0.75 |
| Ex-Smokers, n (%) | | 30 (47) | 4 (24) | 0.19 |
| Never smokers, n (%) | | 18 (28) | 8 (47) | 0.22 |
| Pack year history* | | 8.8 (0.0-24.4) | 0.2 (0.0-16.8) | 0.20 |
| Number taking ICS, n (%) | | 31 (48) | 9 (53) | 0.74 |
| Number of unscheduled primary care and emergency department visits in previous 12 months* | | 2 (0-4) | 1 (0-2) | 0.22 |
| Number of hospital admissions in previous 12 months* | | 0 (0 – 1) | 0 (0 – 1) | 0.96 |
| Heart rate (Beats/Minute)˜ | | 101 (63-142) | 92 (63-133) | 0.13 |
| VAS cough (mm)* | | 49 (23-72) | 53 (35-69) | 0.57 |
| VAS dyspnoea (mm)* | | 73 (38-86) | 70 (0-89) | 0.66 |
| VAS sputum production (mm)* | | 18 (2-50) | 21 (0-31) | 0.75 |
| VAS sputum purulence (mm)* | | 13 (0-52) | 3 (0-31) | 0.34 |
| Height (cm)* | | 167 (161-175) | 163 (155-178) | 0.50 |
| Weight (kg)* | | 78 (69-95) | 83 (63-86) | 1.00 |
| Number of days of worse symptoms in lead-up to exacerbation (days)* | | 4 (2-7) | 3 (1-4) | 0.16 |
| Blood pH* | | 7.4 (7.4-7.4) | 7.4 (7.4-7.5) | 0.15 |
| Temperature (°C)* | | 36.5 (36.1-37.2) | 36.6 (36.4-36.9) | 0.93 |
| SBP (mmHg)* | | 135 (124-147) | 126 (117-141) | 0.18 |
| DBP (mmHg)˜ | | 81 (42-106) | 76 (59-103) | 0.19 |
| Respiratory rate (breaths per minute)* | | 20 (18-24) | 21 (18-22) | 0.81 |
| EuroQol mobility* | | 1 (2-2) | 1 (2-2) | 0.97 |
| EuroQol self-care* | | 1 (1-2) | 1 (1-1) | 0.28 |
| EuroQol usual activity* | | 2 (2-3) | 1 (2-2) | 0.30 |
| EuroQol pain discomfort* | | 2 (1-2) | 2 (1-2) | 0.88 |
| EuroQol anxiety depression* | | 1 (1-2) | 1 (1-2) | 0.86 |
| EuroQol VAS score* | | 33 (9-48) | 30 (19-40) | 0.80 |
| HAD anxiety˜ | | 7 (0-18) | 6 (0-15) | 0.22 |
| HAD depression* | | 4 (2-9) | 5 (2-7) | 0.80 |
| Charlston Index Score * | | 0 (0-1) | 0 (0-2) | 0.18 |
| Neutrophils % ~ | | 70.9 (28.8 – 97.3) | 74.0 (52.2 – 93.2) | 0.36 |
| Lymphocytes % * | | 15.0 (9.7 – 25.3) | 15.5 (8.8 – 19.5) | 0.85 |
| Monocytes % * | | 7.0 (5.3 – 8.4) | 7.9 (5.2 – 8.9) | 0.31 |
| Basophils % * | | 0.5 (0.3 – 0.6) | 0.4 (0.3 – 0.5) | 0.18 |
| Haemoglobin (g/L)˜ | | 143 (109 – 170) | 148 (127 – 163) | 0.11 |
| Platelets (x10⁹cells/L)* | | 258 (222 – 337) | 301 (253 – 374) | 0.11 |
| Sodium (mmol/L)* | | 139 (138 – 141) | 140 (139 – 141) | 0.47 |
| Potassium (mmol/L)* | | 3.8 (3.5 – 4.1) | 3.7 (3.3 – 3.9) | 0.22 |
| Urea (mmol/L)* | | 4.7 (3.7 – 6.3) | 4.6 (4.0 – 6.0) | 0.88 |
| Creatinine (μmol/L)˜ | | 71 (32 - 134) | 68 (37 – 98) | 0.47 |
| eGFR (mL/min)* | | 90 (80 – 90) | 82 (72 – 90) | 0.56 |
| Blood glucose (mmol/L)* | | 6.9 (6.1 – 7.7) | 6.7 (5.9 – 8.4) | 0.89 |
| Serum albumin (g/L)* | | 36 (34 – 38) | 37 (36 – 38) | 0.31 |
| CRP (mg/L)* | | 10.1 (1.7 – 33.3) | 3.9 (1.7 – 19.8) | 0.45 |
| Number of exacerbations associated with increased sputum production, n (%) | | 31 (48) | 10 (59) | 0.45 |
| Number of exacerbations associated with increased sputum purulence, n (%) | | 27 (42) | 5 (29) | 0.34 |
| Number of exacerbations associated with increased cough, n (%) | | 50 (78) | 16 (94) | 0.13 |
| Fever accompanying exacerbation, n (%) | | 33 (52) | 5 (29) | 0.10 |
| Antibiotics in week prior to exacerbation admission, n (%) | | 27 (42) | 6 (35) | 0.61 |
| Hydrocortisone, n (%) | | 8 (13) | 4 (24) | 0.26 |
| Nasal polyps, n (%) | | 6 (9) | 0 (0) | 0.19 |
| Comorbidity, n (%) | CVS | 10 (16) | 5 (29) | 0.43 |
|  | Other | 28 (44) | 6 (35) |  |
|  | None | 26 (41) | 6 (35) |  |
| SABA, n (%) | | 53 (83) | 14 (82) | 0.96 |
| LABA, n (%) | | 5 (8) | 1 (6) | 0.79 |
| Carbocystiene, n (%) | | 4 (6) | 2 (12) | 0.44 |
| Maintenance prednisolone, n (%) | | 5 (8) | 2 (12) | 0.61 |
| Monteleukast, n (%) | | 12 (19) | 4 (24) | 0.66 |
| Antihistamine, n (%) | | 8 (13) | 0 (0) | 0.12 |
| Antihypertensive, n (%) | | 5 (8) | 3 (18) | 0.23 |

*Definition of abbreviations:* ICS = inhaled corticosteroids; SBP = systolic blood pressure; DBP = diastolic blood pressure; CRP = C-reactive protein; CVS = cardiovascular disease; SABA = short-acting beta agonists; LABA = long-acting beta agonists. *Measures of central tendency and spread:* variables marked with the * symbol are presented as median (IQR); variables marked with the ˜ symbol are presented as mean (range). Comparisons between SCS given and SCS not given groups are made using the chi-squared test, wilcox test or t-test as appropriate.

**S2 Table | Random forest importance scores of biological and symptom variables in multivariate SCS prescription prediction models for all participants.**

| **Name of Successive Variable** | **Importance Score of Successive Variable** |
| --- | --- |
| Increased wheeze at exacerbation | 7.0 |
| Eosinophils % | 5.1 |
| Blood pCO2 | -0.7 |
| Oxygen saturation | -2.9 |

Models shown range from those using just a single variable up to those with the full biological and symptom variable subset which passed the univariate analysis filter of p < 0.1. Variable importance scores were calculated as outlined in S1 Appendix Statistical Methods Methods. To illustrate the interpretation of the variable importance scores, consider the example of eosinophils %. The eosinophils % random forest importance score of 5.1 indicates that classification accuracy would drop by 5.1% if eosinophils % is omitted from the classification model.

**S3 Table | Performance of variable subset SCS prescription prediction models for all participants.**

| **Number of Variables in Model** | **AUC of Corresponding Model (2dp)** |
| --- | --- |
| 1 | 0.60 |
| 2 | 0.69 |
| 3 | N/A |
| 4 | N/A |

For a model with x variables, the variables in the model are the top x variables ranked according to variable importance scores in Table 4 of the main manuscript. Note that for the reasons discussed in the S1 Appendix Statistical Analysis, AUCs are only shown for variable subset models for which all included variables in the subset have positive variable importance scores in the full variable model.

**S4 Table | Additional variable comparisons of D0 exacerbation characteristics of patients who failed treatment with those who succeeded treatment.**

| **Characteristic** | **Exacerbation** | | | |
| --- | --- | --- | --- | --- |
|  | **n=81 Events** | | | |
|  | **Treatment Failure, n=43** | | **Treatment Success, n=38** | ***P*-value** |
| Male, n (%) | 14 (33) | | 20 (53) | 0.11 |
| Age (years)* | 54 (30-69) | | 50 (33-63) | 0.53 |
| Asthma, n (%) | 31 (72) | | 28 (74) | 0.70 |
| COPD, n (%) | 12 (28) | | 10 (26) | 0.67 |
| Current smokers, n (%) | 9 (21) | | 12 (32) | 0.27 |
| Ex-Smokers, n (%) | 17 (40) | | 17 (45) |  |
| Never smokers, n (%) | 17 (40) | | 9 (24) |  |
| Pack year history * | 2.8 (0.0-18.4) | | 9.6 (0.2-27.6) | 0.24 |
| Height (cm)* | 165 (160-173) | | 170 (160-178) | 0.26 |
| Weight (kg)* | 80 (67-94) | | 80 (70-94) | 0.76 |
| Number of days of worse symptoms in lead-up to exacerbation (days)* | 4 (2-7) | | 3 (1-7) | 0.47 |
| Blood pH* | 7.4 (7.4-7.4) | | 7.4 (7.4-7.4) | 0.81 |
| Blood pCO2 (kPa)* | 5.7 (5.2-6.1) | | 5.4 (4.7-6.0) | 0.20 |
| Temperature (°C)* | 36.6 (36.2-37.2) | | 36.5 (36.0-37.0) | 0.92 |
| SBP (mmHg)* | 129 (120-143) | | 138 (125-151)) | 0.23 |
| DBP (mmHg)˜ | 81 (42-103) | | 79 (47-106) | 0.65 |
| Heart rate (Beats/Minute)˜ | 102 (63-142) | | 96 (63-132) | 0.15 |
| Respiratory rate (breaths per minute)* | 20 (18-24) | | 20 (18-22) | 0.89 |
| Number of hospital admissions in previous 12 months* | 0 (0 – 1) | | 0 (0 – 1) | 0.34 |
| Number of ITU admissions in previous 12 months* | 0 (0-0) | | 0 (0-0) | 0.82 |
| EuroQol pain discomfort* | 2 (2-2) | | 2 (1-2) | 0.58 |
| EuroQol anxiety depression* | 1 (1-2) | | 2 (1-2) | 0.15 |
| EuroQol VAS score* | 35 (10-45) | | 30 (4-60) | 0.84 |
| HAD anxiety˜ | 7 (0-17) | | 7 (0-18) | 0.93 |
| HAD depression* | 5 (3-9) | | 4 (1-9) | 0.43 |
| Charlston Index Score* | 0 (0-1) | | 0 (0-2) | 0.88 |
| Leucocytes (x10⁹cells/L)* | 10.9 (9.1 – 14.7) | | 10.6 (9.1 – 12.1) | 0.51 |
| Neutrophils (x10⁹cells/L)* | 8.5 (5.9 – 11.0) | | 7.4 (5.4 – 9.3) | 0.38 |
| Eosinophils (x10⁹cells/L)* | 0.13 (0.04-0.33) | | 0.25 (0.08-0.43) | 0.26 |
| Basophils (x10⁹cells/L)˜ | 0.0 (0.0-0.1) | | 0.1 (0.0-0.2) | 0.16 |
| Monocytes (x10⁹cells/L)* | 0.8 (0.6-1.0) | | 0.8 (0.6-0.9) | 0.47 |
| Lymphocytes (x10⁹cells/L)* | 1.7 (1.1-2.0) | | 1.6 (1.1-2.6) | 0.66 |
| CRP (mg/L)* | 10.3 (2.5 – 25.0) | | 7.9 (1.0 – 32.4) | 0.59 |
| Heamoglobin (g/L)˜ | 142 (109-170) | | 146 (115-167) | 0.25 |
| Platelets (x10⁹cells/L)* | 261 (223-302) | | 272 (235-364) | 0.23 |
| Sodium (mmol/L)* | 139 (138-141) | | 139 (137-141) | 0.93 |
| Potassium (mmol/L)* | 3.7 (3.5-4.1) | | 3.8 (3.5-4.2) | 0.35 |
| Urea (mmol/L)* | 4.7 (3.8-6.3) | | 4.5 (3.7-6.0) | 0.59 |
| Creatinine (μmol/L)* | 66 (60-79) | | 70 (58-79) | 0.84 |
| eGFR (mL/min)* | 88 (73-90) | | 90 (79-90) | 0.34 |
| Blood glucose (mmol/L)* | 6.6 (6.1-8.1) | | 7.1 (5.7-7.6) | 0.81 |
| Serum albumin (g/L)* | 36.0 (34.4-37.3) | | 36.7 (35.4-38.0) | 0.24 |
| Number of exacerbations associated with increased cough, n (%) | 34 (79) | | 32 (84) | 0.55 |
| Increased wheeze at exacerbation, n (%) | 37 (86) | | 33 (87) | 0.92 |
| Fever accompanying exacerbation, n (%) | 22 (51) | | 16 (42) | 0.42 |
| SCS in week prior to exacerbation admission, n (%) | 25 (58) | | 21 (55) | 0.79 |
| Antibiotics in week prior to exacerbation admission, n (%) | 19 (44) | | 14 (37) | 0.50 |
| Hydrocortisone, n (%) | 7 (16) | | 5 (13) | 0.69 |
| Nasal polyps, n (%) | 2 (5) | | 4 (11) | 0.31 |
| Comorbidity, n (%) | CVS | 7 (16) | 8 (21) | 0.64 |
|  | Other | 17 (40) | 17 (45) |  |
|  | None | 19 (44) | 13 (34) |  |
| Antibiotics at exacerbation, n (%) | 22 (51) | | 15 (39) | 0.29 |
| LABA, n (%) | 4 (9) | | 2 (5) | 0.49 |
| Carbocystiene, n (%) | 5 (12) | | 1 (3) | 0.12 |
| Maintenance prednisolone, n (%) | 4 (9) | | 3 (8) | 0.82 |
| Monteleukast, n (%) | 10 (23) | | 6 (16) | 0.40 |
| Antihistamine, n (%) | 3 (7) | | 5 (13) | 0.35 |
| Antihypertensive, n (%) | 4 (9) | | 4 (11) | 0.85 |

*Definition of abbreviations:* SBP = systolic blood pressure; DBP = diastolic blood pressure; ITU = intensive therapy unit; CRP = C-reactive protein; CVS = cardiovascular disease; LABA = long-acting beta agonists. *Measures of central tendency and spread:* variables marked with the * symbol are presented as median (IQR); variables marked with the ˜ symbol are presented as mean (range). Comparisons between treatment failure and treatment success groups are made using chi-squared test, wilcox test or t-test as appropriate.

**S5 Table | Performance of variable subset treatment failure prediction models for all participants.**

| **Number of Variables in Model** | **AUC of Corresponding Model (2dp)** |
| --- | --- |
| 1 | 0.62 |
| 2 | 0.68 |
| 3 | 0.64 |
| 4 | 0.64 |
| 5 | 0.66 |
| 6 | 0.67 |
| 7 | 0.69 |
| 8 | 0.73 |
| 9 | 0.76 |
| 10 | 0.80 |
| 11 | 0.81 |
| 12 | 0.81 |
| 13 | 0.81 |
| 14 | 0.80 |
| 15 | 0.81 |
| 16 | N/A |
| 17 | N/A |

For a model with x variables, the variables in the model are the top x variables ranked according to variable importance scores in Table 6 of the main manuscript. Note that for the reasons discussed in the S1 Appendix Statistical Analysis, AUCs are only shown for variable subset models for which all included variables in the subset have positive variable importance scores in the full variable model.

**S1 Appendix References**

1. Breiman L. Random forests. *Mach. Learn*. 2001; 45(1): 5–32.
2. Kuhn, M. (2008). Caret package. Journal of Statistical Software, 28(5).
3. Kuhn M, Johnson K. *Applied Predictive Modeling*. New York: Springer; 2013.
4. Akaike H. A new look at the statistical model identification. *IEEE Transactions on Automatic Control*. 1974; 19(6): 716-723.
5. Schwarz G.  Estimating the Dimension of a Model. *Ann. Statist.* 1978; 6(2) 461-464.
